# Inhibition of STAT3-mediated glycolysis by bruceine D suppresses non-small-cell lung cancer progression *in vitro* and *in vivo*

**DOI:** 10.1080/15384047.2026.2665867

**Published:** 2026-05-08

**Authors:** Yu Zhu, Xuan Xu, Xiaofan Hong, Jiayi Zhang, Yi Zhou, Aofeng Sun, Yihan Ye, Yini Xu, Huanhai Xu, Haiyang Zhao, Chengguang Zhao, Xing Jin, Lehe Yang, Jianxiao Zheng

**Affiliations:** a Affiliated Yueqing Hospital, Wenzhou Medical University, Wenzhou, Zhejiang, China; b The First Affiliated Hospital, Wenzhou Medical University, Wenzhou, Zhejiang, China; c School of Pharmaceutical Sciences, Wenzhou Medical University, Wenzhou, Zhejiang, China; d The Institute of Life Sciences, Wenzhou University, Wenzhou, Zhejiang, China

**Keywords:** Bruceine D, STAT3, NSCLC, glycolysis, inhibitor

## Abstract

**Background:**

Non-small-cell lung cancer (NSCLC) is one of the deadliest malignancies in the world. Signal transducer and activator of transcription 3 (STAT3) plays an important role in the progression of NSCLC. Bruceine D is a bioactive quassinoid compound extracted from *Brucea javanica*, a plant widely used in traditional Chinese medicine.

**Methods:**

Tests of cell function were performed to observe the effects of bruceine D on human NSCLC cell lines (H460, PC-9, and SKMES-1). Levels of STAT3, phosphorylated STAT3, Survivin, and other apoptosis-related proteins were detected via Western blotting. The binding of bruceine D to STAT3 was examined using molecular dynamics simulations and surface plasmon resonance spectroscopy. The effect of bruceine D on STAT3 nuclear localization was examined by immunofluorescence. Furthermore, the glucose uptake, lactate production, and extracellular acidification rates of NSCLC cells were analyzed to confirm that bruceine D inhibited glycolysis in NSCLC. The efficacy of bruceine D *in vivo* was evaluated in a mouse xenotransplantation model.

**Results:**

Bruceine D significantly reduced the viability of NSCLC cells, promoted apoptosis, and inhibited the growth of tumors in a mouse xenograft model. Bruceine D was found to bind directly to STAT3, inhibiting glycolysis in NSCLC cells. Western blotting results suggested that the antitumor effects of bruceine D might be mediated by the inhibition of the phosphorylation and nuclear translocation of STAT3.

**Conclusion:**

Bruceine D exerts a strong inhibitory effect on NSCLC *in vitro* and *in vivo*. Bruceine D has potential application prospects in the field of anti-NSCLC therapy.

## Background

Lung cancer is associated globally with the highest mortality and malignancies.^1^ The global estimates for 2050 predict approximately 3.8 million new cases of lung cancer and 3.2 million deaths due to the disease.^2^ In China, lung cancer incidence mirrors that of the United States, yet the mortality rate is 1.4 times higher, the reasons for which remain elusive.^3^ Non-small-cell lung cancer (NSCLC), comprising approximately 85% of lung cancer cases, shows a rising incidence. Consequently, the development of novel, highly efficacious treatments with low toxicity is imperative to decrease the mortality associated with NSCLC.

*Brucea javanica* (L.) Merr, an evergreen shrub belonging to the Simaroubaceae family, is a material commonly used in traditional Chinese medicines. Its seeds hold therapeutic value in treating a range of ailments, including dysentery, malaria, warts, and corns.^4^ *Brucea javanica* oil has been used clinically for many years as an antitumor drug in China,^5-7^ but its active ingredients have not been clarified. Bruceine D, one of the active ingredients in another extract from *B. javanica,* an ingredient of traditional Chinese herbal medicine, is a water-soluble tricyclic tetraterpenoid compound.^8^ Remarkably, studies have demonstrated bruceine D's robust antitumor activity in multiple cancer types. For example, bruceine D has been shown to activate mitogen-activated protein kinase (MAPK) and c-Jun N-terminal kinase (JNK) signaling pathways through the production of reactive oxygen species (ROS), thus inducing apoptosis and inhibiting the migration activities of breast cancer cells.^9^ Similarly, bruceine D induces apoptosis in pancreatic adenocarcinoma cells by activating P38-MAPK.^10^ Furthermore, bruceine D has also been shown to promote the apoptosis of lung cancer cells.^11^ Consequently, bruceine D emerges as an attractive candidate drug for the prevention or treatment of human cancers.

The signal and activator of transcription (STAT) family of proteins represents a group of important potential molecular targets of anticancer therapies. These proteins, which are also known as the cytoplasmic induced transcription factor family, can transmit extracellular signals across cell membranes to the nucleus for the regulation of expression of target genes. STAT3 is the key member of the STAT protein family, and it regulates the expression of many genes. Importantly, numerous studies have found that STAT3 is an important oncogenic transcription factor responsible for tumor malignant transformation, and its activity has been related to tumor cell proliferation and cancer-related angiogenesis. Additionally, STAT3 has an inhibitory effect on the immune system, thus enhancing its role in the process of tumor cell migration and drug resistance generation.^12^ Aberrant STAT3 activity can lead to the abnormal expression of a variety of downstream genes by transmitting multiple oncogenic tyrosine kinase signals in tumor cells. When cells are stimulated by cytokines or growth factors, they can activate a Janus kinase upstream of STAT3, thus triggering the phosphorylation and dimerization of STAT3 protein to promote entry into the nucleus and the regulation of gene expression.^13^

To meet the high energy demand, cancer cells undergo metabolic changes. The phenomenon of lactate secretion, even in the presence of sufficient oxygen, is known as aerobic glycolysis.^14^ Given the crucial role of aerobic glycolysis in tumor development and progression, targeting this process has emerged as an effective tumor treatment strategy.^15^ STAT3 is vital in promoting aerobic glycolysis, and among its target genes for transcriptional activation are HIF-1α and MYC, which significantly promote aerobic glycolysis.^16^ The phosphorylation of STAT3 results in the overexpression of HIF-1α and MYC, thereby inducing aerobic glycolysis. Research indicates that STAT3 not only mediates the expression of HIF-1α but also binds to the promoters of its target genes to form a transcription complex, facilitating the expression of HIF-1α target genes.^17^ Furthermore, STAT3 can directly regulate the transcription of glycolysis-related enzymes.^18^ These studies further demonstrate that STAT3 plays a critical role in tumor glycolysis.

In this study, we evaluated the effects of bruceine D on NSCLC cells *in vitro* and *in vivo*, aiming to test the hypothesis that bruceine D may partially inhibit the growth, migration, and metabolism of NSCLC cells by downregulating the activation of STAT3. The results of this study provide an experimental basis for the activity of bruceine D in the inhibition of malignancy of NSCLC cells, and this study suggests that further investigations into potential applications of bruceine D in the prevention and treatment of NSCLC are warranted.

## Materials and methods

### Cell culture

The human cell lines BEAS-2B (CBP60577), H460 (CBP60138), and SKEMS-1 (CBP60152) were purchased from the Cell Resources Center of the Shanghai Institutes for Biological Sciences (Chinese Academy of Sciences, Shanghai, China). PC-9 cells (SCSP-5085) were purchased from National Collection of Authenticated Cell Cultures. H460 cells, PC-9 cells, BEAS-2B cells, and SKMES-1 cells were grown in RPMI-1640 media (Thermo Fisher Scientific, Waltham, MA, USA, 12633020), Dulbecco’s modified Eagle's medium (DMEM) (Thermo Fisher Scientific, Waltham, MA, USA, 11965092)(PC-9 and BEAS-2B) and minimum essential medium (MEM) (Thermo Fisher Scientific, Waltham, MA, USA, 11095080), respectively. All culture media were supplemented with 10% fetal bovine serum (FBS) (Gibco, NY, USA, A5256701), and all the cells were incubated at 37 °C with 5% CO_2_. Bruceine D (CAS#: 21499-66-1) was purchased from Baoji Herbest Bio-Tech Co., Ltd. and was dissolved in DMSO. Napabucasin (CAS#:HY-13919) was purchased from MCE.

### Cell transfection

Cells were passaged and seeded into 6-well plates at a density of 2 × 10⁴ cells per well. When the cell confluence reached approximately 60%, transfection was performed. For preparation of the transfection complexes, 1 μg of the overexpression plasmid was diluted in 250 μL of Opti-MEM (11058021, Gibco) containing 5 μL of Lipofectamine 3000 and incubated for 5 min. Separately, 5 μL of P3000 reagent was diluted in 250 μL Opti-MEM containing 5 μL of Lipofectamine 3000 and incubated for 5 min. After washing the cells with phosphate-buffered saline (PBS), the transfection complexes were added, and fresh culture medium was supplemented to a final volume of 2 mL per well. Cells were then incubated for 48 h, after which the transfection efficiency was assessed, and only samples meeting the required efficiency criteria were used for subsequent experiments. The transcript number of STAT3 used is NM_139276.3. Lipofectamine 3000 (L3000015) was purchased from Thermo Fisher.

### MTT assay

Cells were inoculated in 96-well plates (3 × 10^3^ cells/well) with 100 µL of the appropriate medium and incubated overnight to allow attachment. Bruceine D was added to the wells, and the plates were incubated for 48 h. The cells were then treated with 25 µL/well MTT solution (5 mg/mL) for 4 h at 37 °C. The formazan crystals were dissolved in 150 µL of DMSO (MCE, HY-Y0320C), and the optical density (OD) was measured at 490 nm using a microplate reader. Half-maximal inhibitory concentrations (IC_50_) were determined using GraphPad Prism 7.0. Methylthiazolyldiphenyl-tetrazolium bromide (MTT) was purchased from YEASEN, Shanghai, China (Document Number: 40201ES72).

### Colony formation assay

Cells were added to 6-well plates (800 cells/well) and left to attach overnight. Bruceine D or DMSO was added to the wells, and the plates were incubated for 2 to 6 h prior to replacing of the medium with fresh culture medium. After 1 week of culture, the colonies were fixed with 4% paraformaldehyde (Merck, Germany, Document Number: 441244) for 15 min and stained with 1% crystal violet for 10 min. Excess stain was removed with PBS, and the plates were allowed to air dry. Colonies were counted via visual observation. Crystal violet was purchased from YEASEN, Shanghai, China (Document Number: 60505ES25).

### EdU assay

EdU assays were performed using a commercially available kit (Biyuntian; CAS#:C0075S) according to the manufacturer's instructions. Briefly, cells were inoculated into 6-well plates and incubated overnight in the appropriate medium. After the addition of bruceine D and an additional 24 h incubation, the cells were incubated with the EdU reagent for 1 h at 37 °C. The cells were fixed, washed, and incubated with AlexaFluor 555. Signal was observed with a confocal fluorescent microscope (Olympus FLUOVIEW FV3000) using 555/580 nm filters. The ratio of EdU-positive cells to the total number of cells was calculated, followed by normalization and subsequent statistical analysis.

### Assessment of cell apoptosis by flow cytometry

Apoptosis was detected with FITC Annexin V Apoptosis Detection Kit I (BD Biosciences, USA, Document Number: 556547). In brief, cells were inoculated in 6-well plates and treated with DMSO or bruceine D for 24 h. Cells were collected and treated according to the instructions of the apoptosis kit, then were incubated with fluorescein-labeled annexin V and propidium iodide (PI). After fluorescence compensation, 10,000 cells were selected from each sample for analysis. Apoptosis assessment was performed with a NovoCyte Opteon flow cytometry system (NovoCyte 2040R ACEA). Data were analyzed using NovoExpress software (Version 1.6.2; Agilent Technologies, Santa Clara, CA, USA). During the analysis, the cells were initially gated based on the FSC-H and SSC-H parameters to define the primary cell population (P1). Subsequently, within the P1 gate, FSC-H and FSC-A parameters were used to exclude doublets and aggregates, thereby identifying the single-cell population (P2). Apoptosis rates were then analyzed within the P2 population.

### Cell migration assays

Cell migration assays were performed using a Transwell filter system (BD Biosciences, United States). Cells were seeded in the upper chamber and incubated in serum-free media. Media containing 10% FBS was added to the lower chamber. After treatment with bruceine D or DMSO for 48 h, the cells were fixed with 4% paraformaldehyde, and nonmigrated cells were removed from the upper surface of the filter. Migrated cells were stained with 1% crystal violet for 10 min and were observed using a light microscope.

### Western blotting

Cells were lysed in lysis buffer, the supernatant was obtained by centrifugation, and equal amounts of soluble protein were separated by SDS-PAGE using 10% or 12% acrylamide gels. Proteins were transferred electrophoretically to PVDF membranes, which were blocked with 5% nonfat milk for 2 h. The membranes were incubated with specific primary antibodies for 6 h at 4 °C. After washing, the membranes were incubated with the appropriate secondary antibodies for 1 h. Antibody staining was visualized using Omni-ECL Femto Light Chemiluminescence Kit (EpiZyme, Shanghai, China), and the images were analyzed with Image J.

The following primary antibodies were obtained from commercial sources: anti-GAPDH (AB-P-R001, GoodHere Technology), anti-P-STAT3 (phospho Y705; ab76315, Abcam), anti-STAT3 (#12640, Cell Signaling Technology), anti-Bax (ab32503, Abcam), anti-Bcl-2 (sc-56015, Santa Cruz, CA, USA), anti-Cleaved PARP1 (ab32064, Abcam), anti-N-cadherin (ab18203, Abcam), anti-Snail (C15D3, Cell Signaling Technology), anti-Vimentin (ab137321, Abcam), Lamin B1 (#12586, Cell Signaling Technology), Ki-67 (#9027, Cell Signaling Technology) and caspase-3 (#9661, Cell Signaling Technology).

### Immunofluorescent staining

Cells were inoculated into 6-well plates and incubated overnight. Subsequently, the cells were exposed to the indicated compounds for 24 h and then cultured in medium B for a further 4 h before the addition of IL-6 for 1 h. After fixing the cells with 4% paraformaldehyde (Merck, Germany, Document Number: 441244), they were incubated with 1% Triton X-100 (Merck, Germany, Document Number: 93443) for 15 min and 1% BSA for 1 h. The cells were then incubated with anti-P-STAT3 (phospho Y705; ab76315; Abcam) in 4% BSA for 6 h and then with a secondary antibody for 1 h. The nuclei were stained with DAPI, and the samples were observed under a fluorescent microscope. Recombinant human IL-6 was purchased from Bio-Techne China Co., Ltd. (206-IL-010).

### Molecular docking

Molecular docking was performed using AutoDock Vina 1.0.2 software. Data representing the crystal structure of STAT3 (PDB code: 6NJS) were obtained from the Protein Data Bank. The input files of the ligand and receptor were generated using AutoDock Tools 1.5.6 software (The Scripps Research Institute, CA, USA). During the docking process, the protein was considered rigid, and the ligand was flexible.

### Molecular dynamics

This study conducted molecular dynamics simulations using Gromacs 2022 software. Force field parameters were obtained using the pdb2gmx tool built into Gromacs and the AutoFF web tool, respectively: among them, the amber14sb force field was adopted for the molecular parameters of the receptor protein, while the GAFF2 force field was selected for the molecular parameters of the ligand. To achieve solvation of the system, a 1 nm TIP3P-type cubic water box was added around the system^19^; ions were introduced into the system using the gmx genion tool to bring the system to an electrically neutral state.​

Long-range electrostatic interactions were handled using the Particle Mesh Ewald (PME) method, with a cutoff distance set to 1 nm; constraints on all chemical bonds were implemented via the SHAKE algorithm, and the integration time step for molecular dynamics simulations was set to 1 fs using the Verlet leapfrog algorithm. Prior to the initiation of molecular dynamics simulations, energy optimization of the system was required: the energy minimization process was divided into two steps, starting with 3,000 steps of steepest descent optimization, followed by 2,000 steps of conjugate gradient optimization. The specific optimization procedure was as follows: first, constrain the solute and perform energy minimization on water molecules; second, constrain the counterions and conduct energy minimization; finally, complete energy minimization of the entire system under unconstrained conditions.​

The simulation was run under the conditions of a temperature of 310 K and an NPT system under constant pressure, with a simulation duration of 100 ns. During the simulation, the g-rmsd, g-rmsf, g-hbond, g-Rg, and g-sasa tools were used to calculate the root mean square deviation (RMSD), root mean square fluctuation (RMSF), hydrogen bonds (HBonds), radius of gyration (Rg), solvent-accessible surface area (SASA), and Gibbs free energy, respectively; the MM-PBSA binding free energy of the complex was calculated using the g_mmpbsa package in GROMACS.

### Glycose consumption and lactic acid production

NSCLC cells were seeded in 24-well plates (2 × 10^4^) and cultured until adherence was achieved. The medium was then replaced, and the initial glucose and lactic acid concentrations were measured. After 48 h of culture, the medium was collected and centrifuged at 12,000 rpm for 10 min at 4 °C to measure the glucose and lactate concentrations. The glucose or lactate concentration was determined using a glucose assay kit (Jiancheng, Nanjing, China, Document Number: A019-2-1) and a lactate assay kit (Jiancheng, Nanjing, China, Document Number: A154-1-1), respectively, following the manufacturer's instructions. Glucose consumption and lactate production over 48 h were calculated based on the difference between initial and final concentrations according to a standard curve.

### Detection of glycolysis

The glycolytic capacity was evaluated using the Seahorse XF Glycolytic Rate Assay Kit (Agilent Technologies, USA). One hour before the assay, the cells were incubated with XF RPMI medium (containing HEPES) with 2 mM glutamine, 10 mM glucose, and 1 mM pyruvate at 37 °C in a CO_2_-free incubator. Analysis was performed by adding 0.5 µM rotenone/antimycin A (Rot/AA) and 50 mM 2-deoxy-D-glucose (2-DG). Basal and compensatory glycolysis rates were assessed, taking into account the contribution of CO_2_ to the extracellular acidification induced by mitochondrial respiration.

### SPR analysis

The OpenSPRTM instrument standard operating procedures were followed to install the NTA chip. Running at the maximum flow rate (150 µL/min) with the assay buffer PBS, and adjust the flow rate of the buffer to 20 µL/min after reaching a stable signal baseline. Inject the prepared imidazole (Merck, Germany, Document Number: 56749) and NiCl_2_ (Merck, Germany, Document Number: 339350) solution through the inlet port to complete the chip surface functionalization. Prepare 200 μL of solubilized ligand protein. Flow rate was 20 µL/min, and the binding time was 4 min. Observe baseline for 5 min to ensure stability. After the ligand signal is stabilized, start injecting a high concentration of analyte to confirm the ligand activity and confirm the approximate maximum binding capacity of the surface. Increase the flow rate to 150 µL/min and inject the appropriate regeneration buffer to remove the analyte.

Dilute the analyte with buffer and load sample at 30 µL/min. The binding time of protein and ligand was 60 s; the time of natural dissociation time was 120 s. The analysis software used for the results was GraphPad Prism7, and the analysis method was the Steady State Affinity analysis model.

### Xenograft models

All protocols for the animal experiments were approved by the Animal Policy and Welfare Committee of Wenzhou Medical University. All animals used in this study were maintained under specific pathogen–free (SPF) conditions at the Laboratory Animal Center of Wenzhou Medical University. BALB/c nude mice(Congenital Thymus Absence Immunity) weighing 18–20 g were acclimated for 1 week prior to experimentation. The study was terminated immediately when the tumor volume reached or approached one-tenth of the mouse body weight (i.e., ~10% tumor burden). A mixture of H460, PBS, and Matrigel was implanted into the hind abdomen of nude mice. The longest tumor diameter was defined as the length (*L*), and the diameter perpendicular to the longest diameter was defined as the width (*W*). Tumor volumes were measured as *V* = (*L* × *W* × *W*)/2. When the tumor volume was appropriate, 32 mice were randomly divided into four groups and injected intraperitoneally with napabucasin or bruceine D. Eight mice in the control group were treated with PBS, eight mice were treated with napabucasin (2.5 mg/kg), eight mice were treated with a low dose (1 mg/kg) of bruceine D, and eight mice were treated with a high dose of bruceine D (2.5 mg/kg). Every 2 d, the tumor volumes were measured, the mice were weighed, and additional doses were given. At the end of the experiment, the mice were euthanized in a compliant sealed chamber using a gradual-fill CO₂ method. CO₂ was introduced until the animals became unconscious, followed by continued exposure for at least 1 min. Death was confirmed by the absence of respiration and cardiac activity, together with pupil dilation. The tumors were excised, and the hearts, livers, kidneys, and lungs were preserved in 4% paraformaldehyde for histological and protein expression analyses. The *t* test method was used to analyze the final tumor weights of each group. Tumor volume and mouse weight over time were analyzed by two-way repeated-measures ANOVA with treatment and time as factors.

### Hematoxylin and eosin staining

The key organs (heart, liver, lung, and kidney) were harvested and fixed in 4% paraformaldehyde, followed by paraffin embedding. Paraffin-embedded tissues were sectioned at a thickness of 5 μm, deparaffinized in xylene, and rehydrated through a graded ethanol series, after which the sections were sequentially stained with hematoxylin (Merck, Germany, Document Number: H3136) and eosin (Merck, Germany, Document Number: 31890) for the evaluation of histomorphological changes.

### Immunohistochemistry

Mouse xenograft tumors were collected and fixed within 30 min in 4% paraformaldehyde (Merck, Germany, Document Number: 441244) for 18–24 h, followed by dehydration, xylene (Merck, Germany, Document Number: 534056) clearing, paraffin embedding, and sectioning at 4–5 μm. After deparaffinization and rehydration, the sections underwent citrate buffer–based heat-induced antigen retrieval, endogenous peroxidase blocking with 3% hydrogen peroxide (Merck, Germany, Document Number: 88597), and nonspecific blocking, followed by incubation with primary and secondary antibodies. Signal was developed using DAB (Merck, Germany, Document Number: D8001), counterstained with hematoxylin, dehydrated, mounted, and examined under a light microscope. The following primary antibodies were obtained from commercial sources: anti-P-STAT3 (phospho Y705; ab76315; Abcam), Ki-67 (9027; Cell Signaling Technology), and caspase-3 (#9661; Cell Signaling Technology). Immunohistochemical staining was quantitatively analyzed using ImageJ software (version 1.54 g; National Institutes of Health, Bethesda, MD, USA). After color deconvolution, the images were converted to grayscale, and the integrated optical density (IOD) and positive staining area were measured. The relative expression level of the target protein was calculated as the IOD/area. The corresponding description has been added to the Methods section of the revised manuscript.

### Statistical analysis

Data are presented as the mean ± standard deviation (SD) of three parallel (independent) experiments. Between-group differences were assessed using unpaired, two-tailed Student's *t* tests in GraphPad Prism (version 7). Tumor volume and mouse weight over time were analyzed by two-way repeated-measures ANOVA, with treatment and time as factors. A *p* value <0.05 was considered statistically significant.

## Results

### Bruceine D decreases the viability of NSCLC cells

**Figure 1.**
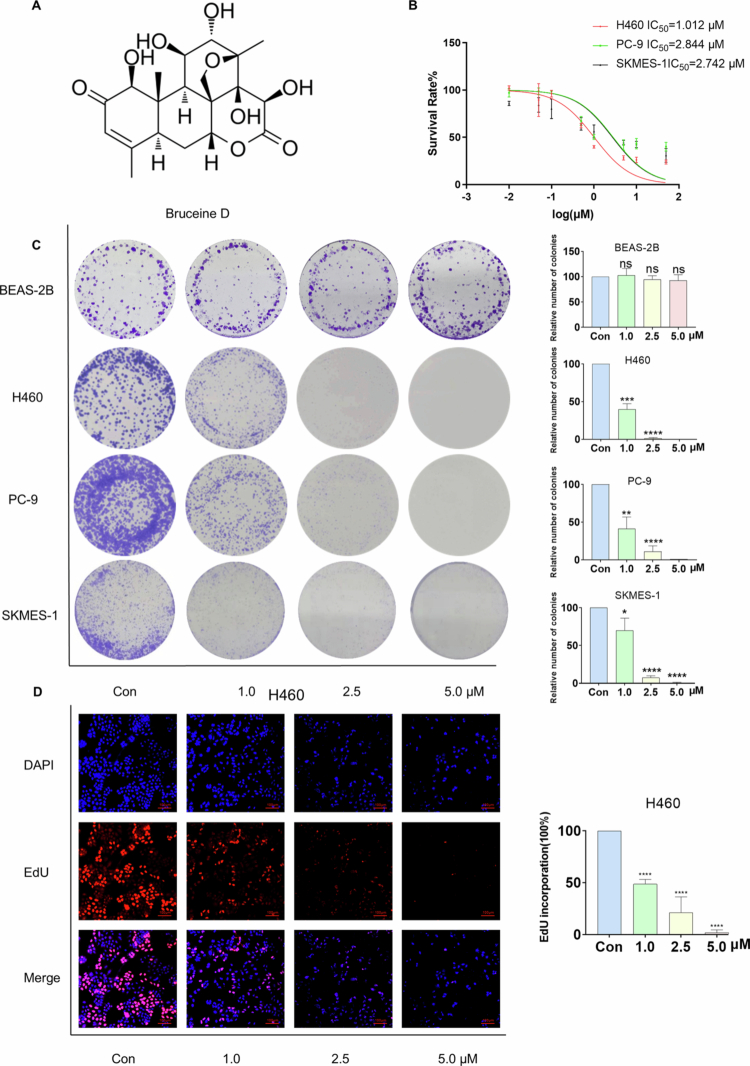
Bruceine D inhibited NSCLC cell proliferation. (A) Chemical structure of bruceine D. (B) NSCLC cell and BEAS-2B viability assessed by MTT assay after bruceine D treatment for 48 h, and the IC_50_ value is indicated. (C) NSCLC cells and BEAS-2B were incubated in bruceine D-containing medium for 24 h and allowed to form colonies for 1–2 weeks. (D) After treating H460 with bruceine D for 24 h, Edu reagent was added to detect the proliferation of H460. In the figure, red indicates positive cells and blue indicates DAPI staining (100× magnification). Data were presented from at least three independent experiments (*p < 0.05, **p < 0.01, ***p < 0.001, and ****p < 0.0001).

We investigated the inhibitory effect of bruceine D (Figure 1A) on the proliferation of NSCLC cells and BEAS-2B. In the MTT assays, treatment with bruceine D resulted in a dose-dependent inhibition of the viability of all three NSCLC cell lines (H460, PC-9, and SKMES-1). The IC_50_ values characterizing the inhibition of viability of H460, PC-9, and SKMES-1 cells were 1.012 ± 0.1338, 2.844 ± 1.846, and 2.742 ± 1.443 µM, respectively (Figure 1B), and the IC_50_ of bruceine D in BEAS-2B was >100 μM, indicating low cytotoxicity (Supplementary Figure 1A). Similarly, except for BEAS-2B cells, the colony formation ability of all three types of NSCLC cells decreased significantly with increasing bruceine D concentration (Figure 1C). These findings indicate that bruceine D effectively inhibits the proliferation of NSCLC cells, while exhibiting no detectable cytotoxicity toward normal cells. EdU assays showed that bruceine D significantly inhibited the proliferation of NSCLC cells in a dose-dependent manner (Figure 1D, Supplementary Figure 1B). Taken together, these data indicated that treatment with bruceine D inhibits the viability of NSCLC cells.

### Bruceine D induces apoptosis in NSCLC cells

**Figure 2.**
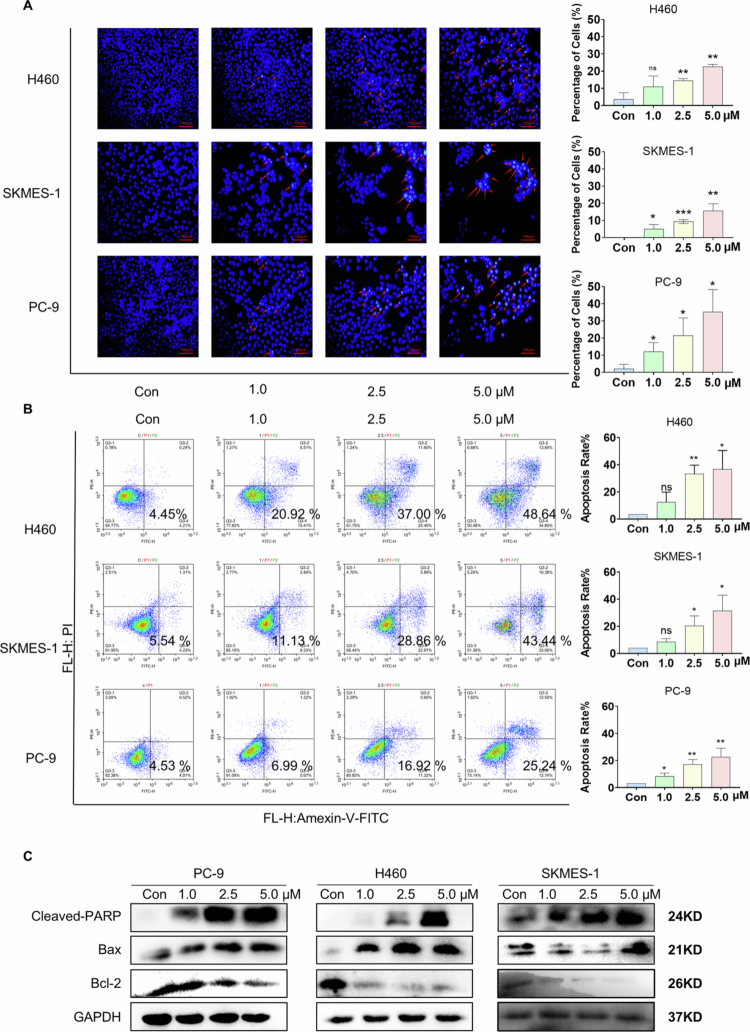
Bruceine D induces apoptosis in NSCLC cells. (A) NSCLC cells were stained with Hoechst 33342 and observed under a fluorescence microscope (100× magnification). The arrows show cells that are clearly highlighted. (B) After bruceine D treatment for 48 h, H460, PC-9, and SKMES-1 cells were collected, and apoptotic cells were analyzed by flow cytometry. Statistical analysis of the percentage of apoptotic cells is shown. (C) Western blotting assays measured the expressions of Bcl-2, Bax, and Cleaved-PARP1 in H460, PC-9, and SKMES-1 cells after 24 h of treatment with bruceine D. (*p < 0.05, **p < 0.01, ***p < 0.001, and ****p < 0.0001).

To explore whether bruceine D reduces the viability of NSCLC cells by inducing apoptosis, the cells were treated with bruceine D for 24 h and then the DNA was visualized by staining with Hoechst 33258. Cells treated with bruceine D fluoresced strongly and demonstrated DNA staining patterns that are consistent with apoptosis (Figure 2A). Next, we stained the three cell lines with annexin V-FITC and PI following 24 h of treatment with three different concentrations of bruceine D, and we detected the percentage of apoptotic cells via flow cytometry. This experiment similarly demonstrated that bruceine D could induce the apoptosis of NSCLC cells (Figure 2B).

In order to investigate the induction of apoptosis by bruceine D at the molecular level, we used Western blotting to examine changes in the levels of apoptosis-related proteins upon treatment with the compound. Here, we found that treatment of NSCLC cells with bruceine D resulted in increased expression of the proapoptotic protein Bax, decreased expression of the antiapoptotic protein Bcl-2, and increased caspase-mediated cleavage of PARP1 (Figure 2C, Supplementary Figure 2). Together, these data suggest that bruceine D induces apoptosis in NSCLC cell lines.

### Bruceine D induces loss of migration potential in NSCLC cells

**Figure 3.**
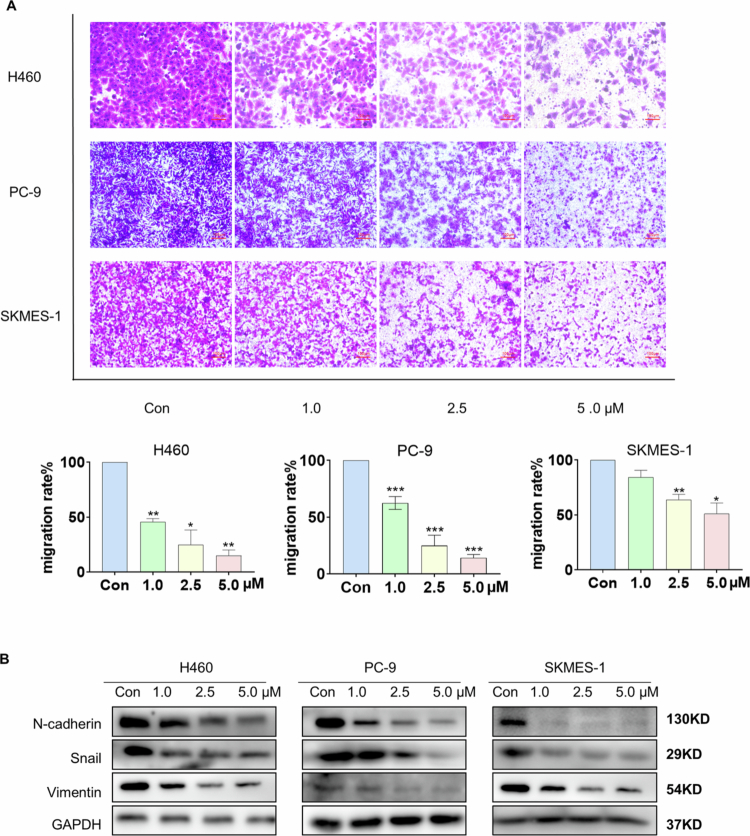
Bruceine D induced migration of NSCLC cells. (A) NSCLC cells were seeded in upper Transwell chambers and treated with bruceine D for 24 h. The migrating cell number was determined (100× magnification). (B) Western blotting assays measured the expressions of N-cadherin, Snail, and Vimentin in H460, PC-9, and SKMES-1 cells after 24 h of treatment with bruceine D. (*p < 0.05, **p < 0.01, ***p < 0.001, and ****p < 0.0001).

We next used Transwell assays to evaluate whether, in addition to viability, bruceine D affects the migration ability of cancer cells. As shown in Figure 3A, we found that, as compared with the control group, cells treated with bruceine D exhibited lower migration, and the number of cells that migrated through the filter decreased significantly with increasing bruceine D concentration (the migrated cells in the figure are stained purple). We also examined the effects of bruceine D treatment on the expression of migration-associated proteins. As shown in Figure 3B and Supplementary Figure 3, the levels of expression of N-cadherin, Snail, and Vimentin in the tumor cells were significantly reduced after bruceine D treatment. Therefore, we concluded that bruceine D inhibits the migration of NSCLC cells in a dose-dependent manner.

### Bruceine D is capable of interacting with STAT3 to form a stable complex

**Figure 4.**
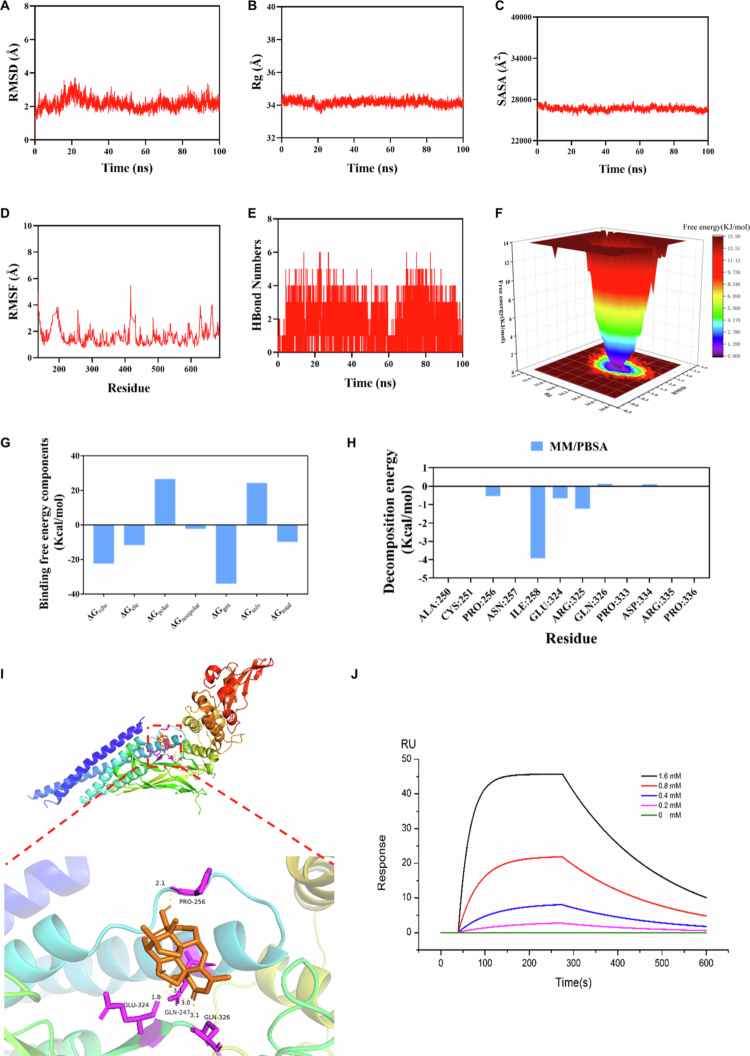
Bruceine D can bind to STAT3. (A) RMSD of the MD simulation of the bruceine D and STAT3 complex systems. (B) Rg of the MD simulation of the bruceine D and STAT3 complex systems. (C) SASA of the MD simulation of the bruceine D and STAT3 complex systems. (D) RMSF of the MD simulation of the bruceine D and STAT3 complex systems. (E) HBond numbers. (F) FEL of the MD simulation of the bruceine D and STAT3 complex systems. (G) Calculation of the binding free energy between bruceine D and STAT3 using the MM/PBSA method. (H) Residue contribution. (I) Molecular docking of bruceine D binding to the STAT3 crystal structure. (J) SPR test was used to detect the binding energy of bruceine D and STAT3.

Due to the importance of STAT3 in NSCLC cells, we hypothesized that bruceine D can exert its effects on NSCLC cell lines in part through binding to STAT3. In order to investigate this conjecture, we used software to perform a molecular dynamics simulation. The results of the molecular dynamics simulation indicate that the compound bruceine D exhibits a strong interaction with STAT3. The simulation was evaluated using RMSD, which showed that the complex reached equilibrium after 95 ns, with fluctuations ultimately stabilizing around 2 Å (Figure 4A). Throughout the simulation, the radius of gyration (Rg) and solvent-accessible surface area (SASA) of the complex exhibited minimal fluctuations (Figure 4B, C), while the RMSF values remained relatively low (mostly below 3 Å) (Figure 4D), suggesting high stability of the complex. Hydrogen bonds play a crucial role in ligand‒protein binding, and the simulation results demonstrate that, in most cases, compound bruceine D can form four hydrogen bonds with STAT3 (Figure 4E), indicating favorable hydrogen bond interactions between the ligand and the target protein. The free energy landscape (FEL) illustrates the free energy distribution based on the RMSD and Rg during the molecular dynamics simulation of STAT3 and the compound bruceine D (Figure 4F). Subsequently, the binding free energy between compound bruceine D and STAT3 was calculated using the MM/PBSA method, yielding a value of −9.88 kcal/mol (Figure 4G). Further analysis of the key amino acid residues in STAT3 involved in binding revealed that ILE258, ARG325, GLU324, and PRO256 play important roles in stabilizing the complex (Figure 4H). The molecular docking simulation offers a more direct visual representation of how bruceine D binds to STAT3 (Figure 4I). This potential binding interaction was investigated *in vitro* using SPR spectroscopy, and STAT3 and bruceine D were found to physically interact with an affinity constant of 2.14 μM (Figure 4J). It was close to the IC_50_ of PC-9 and SKMES-1.

### Bruceine D blocks the activation of STAT3

**Figure 5.**
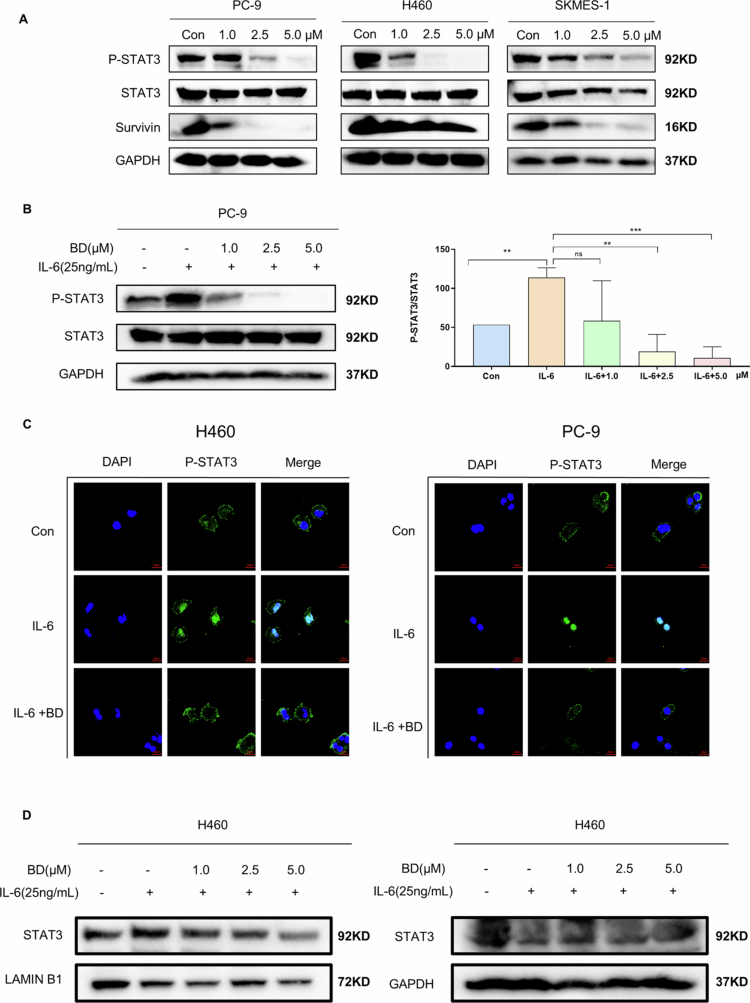
Bruceine D blocks the activation of STAT3. (A) NSCLC cells were treated with bruceine D for 24 h, and Western blotting assays measured the expressions of P-STAT3, STAT3, Survivin, and GAPDH. (B) After PC-9 cells were treated with bruceine D for 24 h, IL-6 was added for 1 h, and Western blotting assays measured the expressions of P-STAT3, STAT3, and GAPDH. A statistical graph is shown on the right. (C) Microscopic images indicating the localization of P-STAT3 (green) and DAPI in H460 and PC-9 cells (600× magnification). (D) After H460 cells were treated with bruceine D for 24 h, IL-6 was added for 1 h, and Western blotting assays measured the expressions of STAT3 in the nucleus and cytoplasm. (*p < 0.05, **p < 0.01, ***p < 0.001, and ****p < 0.0001).

We next attempted to determine whether bruceine D inhibits the phosphorylation and activation of STAT3 in NSCLC cells. In all three NSCLC cell lines, Western blotting analyses demonstrated that the levels of phosphorylated STAT3 (P-STAT3) and the downstream protein Survivin decreased in a time- and dose-dependent manner (Figure 5A, Supplementary Figure 4A). To further explore the effect of bruceine D on STAT3 activity, the amount of P-STAT3 produced upon stimulation with IL-6 was investigated in PC-9 cells. After being treated with bruceine D, the amount of P-STAT3 with IL-6 stimulation was reduced in a dose-dependent manner (Figure 5B, Supplementary Figure 4B).

Because STAT3 activation via phosphorylation leads to its entry into the nucleus, here, serum-starved H460, PC-9, and SKMES-1 cells were treated with bruceine D at a concentration of 5.0 μm. After 24 h of incubation, these cells were stimulated with IL-6 for 30 min and fixed. Immunofluorescence demonstrated that bruceine D blocked the entry of P-STAT3 into the nucleus (Figure 5C, Supplementary Figure 5). STAT3 migrated into the nucleus after IL-6 stimulation, but the result of Western blotting was shown bruceine D inhibited this migration (Figure 5D). Our results show that IL-6 stimulation leads to the nuclear localization of most of the STAT3, but the amount of P-STAT3 and the amount of STAT3 entering the nucleus were significantly reduced after bruceine D treatment. These data suggest that bruceine D can bind to STAT3 and inhibit the phosphorylation and nuclear entry of STAT3. Therefore, the effect of bruceine D against NSCLC cells may be achieved by affecting the activity of STAT3.

### Bruceine D inhibits the glycolytic capacity of NSCLC cells

**Figure 6.**
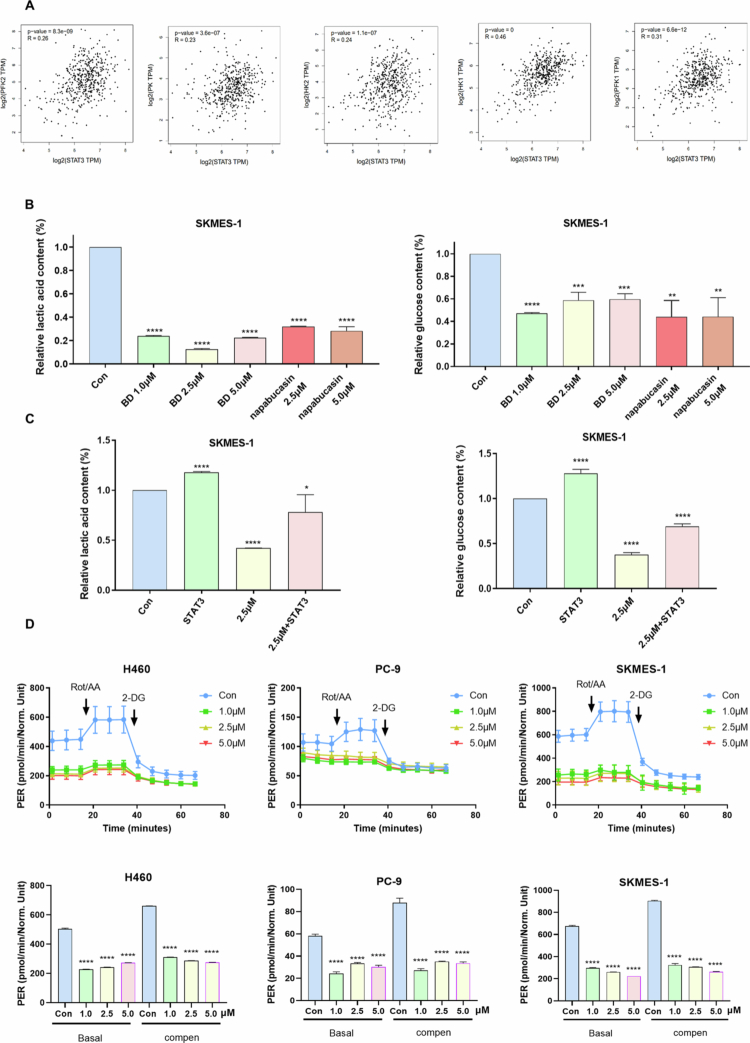
Bruceine D inhibited glycolysis in NSCLC. (A) Analysis of correlations of STAT3 with PFK1, PFK2, PK, HK1, and HK2. (B) SKMES-1 treated with bruceine D or NAPABUCASIN for 48 h, detection of glucose consumption and lactic production in the control or treatment group. (C) When STAT3 was overexpressed, SKMES-1 was treated with bruceine D (2.5 μM) for 48 h, detection of glucose consumption and lactic production in the control or treatment group. (D) Glycolytic proton flow rate of NSCLC cells treated with the treatments of different concentrations of bruceine D. (*p < 0.05, **p < 0.01, ***p < 0.001, and ****p < 0.0001).

STAT3, a critical transcription factor in tumor development, is closely associated with the proliferation, migration, drug resistance, and metabolism of NSCLC. Recent studies suggest that STAT3 can regulate glycolysis in hepatocellular carcinoma, leading to the hypothesis that bruceine D could inhibit NSCLC's glycolytic capacity by targeting STAT3. Initially, the correlation between STAT3 and key glycolytic enzymes was explored using the GEPIA database, revealing a positive correlation between STAT3 and phosphofructokinase 2, pyruvate kinase, hexokinase 1, and hexokinase 2 (Figure 6A). Subsequent testing of glucose uptake and lactate production in H460, PC-9, and SKMES-1 cell lines demonstrated that treatment with bruceine D for 48 h resulted in a significant reduction in both parameters, which was comparable to the effects observed with the STAT3 inhibitor napabucasin (Figure 6B, Supplementary Figure 6A‒C). These findings indicate that the inhibition of STAT3 suppresses basal metabolic activity in NSCLC. Furthermore, enforced overexpression of STAT3 in NSCLC cells followed by bruceine D treatment restored glucose uptake and lactate production (Figure 6C, Supplementary Figure 6D). To further examine the impact of bruceine D on glycolysis in NSCLC, the glycolysis rate kit was employed to measure the rate of glycolytic proton flow after 48 h of treatment. Findings showed a significant reduction in glycolytic capacity following bruceine D treatment (Figure 6D), suggesting that bruceine D can impair cell glycolysis by inhibiting STAT3 activation, with this inhibitory effect appearing to be independent of the bruceine D dosage used.

### Bruceine D inhibits tumor growth in an NSCLC xenograft model

**Figure 7.**
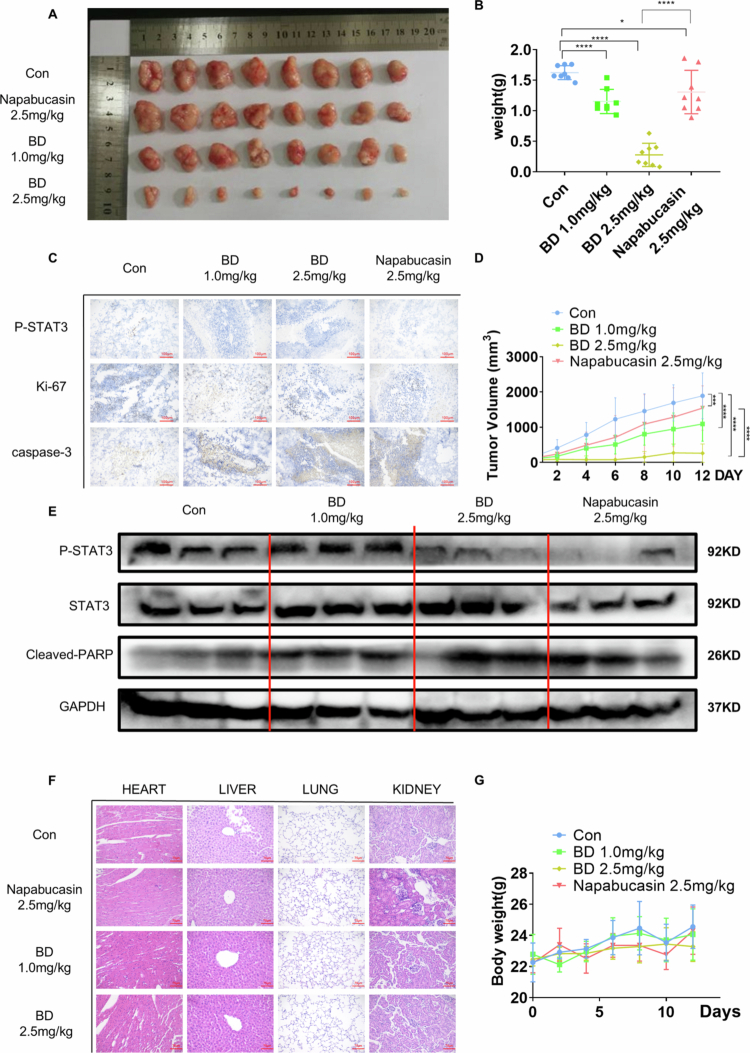
Bruceine D inhibited the growth of the NSCLC xenograft model. (A) The appearance of tumors isolated from the mouse xenograft model. (B, D) Tumor volume and tumor mass in nude mice treated with bruceine D or napabucasin. (C) Immunohistochemical detection of the expression levels of Ki67, P-STAT3, and Caspase-3 in tumor tissues (100× magnification). (E) The levels of P-STAT3 and Cleaved PARP1 in tumor tissue were monitored by western blotting analysis. (F) Representative photomicrographs of sections of livers, hearts, lungs, and kidneys in nude mice (200× magnification). (G) Body weight curves of nude mice treated with bruceine D or napabucasin. (*p < 0.05, **p < 0.01, ***p < 0.001, and ****p < 0.0001).

A mouse xenograft model was developed by subcutaneously administering H460 cells to BALB/c nude mice. This model was used to determine whether bruceine D can inhibit the growth of cancer cells *in vivo*. The positive control was treatment with the STAT3 inhibitor napabucasin. The results showed that intraperitoneal injections of 1 and 2.5 mg/kg bruceine D led to a reduction in tumor volume and weight as compared with both negative control mice and mice treated with napabucasin (Figure 7A, B, D). At the same dose, the efficacy of bruceine D was significantly better than napabucasin. In the tumors of mice treated with bruceine D, we found that STAT3 phosphorylation was inhibited, and the production of Cleaved PARP1 was increased (Figure 7E). The results of immunohistochemistry analyses showed that the levels of both STAT3 and Ki-67 were decreased upon treatment with bruceine D, and the level of cleavage of caspase-3 was increased (Figure 7C).

These results suggested that bruceine D induces the apoptosis of cancer cells *in vivo*. Importantly, no weight loss was observed in any of the bruceine D treatment groups (Figure 7G). The toxicity of bruceine D to the mice was evaluated using hematoxylin and eosin (H&E) staining analyses of the heart, liver, lung, and kidney. No significant cellular inflammation, edema, or necrosis was observed, suggesting that bruceine D has a good safety profile (Figure 7F). Taken together, these results suggested that bruceine D can inhibit the growth of tumors and induce the apoptosis of tumor cells *in vivo*. Thus, we conclude that bruceine D has potent activity against the growth of implanted NSCLC tumors while exhibiting minimal toxicity to the animal.

## Discussion

In this study, we investigated the anticancer activity of bruceine D. The fruit and leaves of Brucea have been commonly used in medicinal preparations. Modern pharmacological studies have shown that extracts from Brucea have both antitumor and anti-inflammatory activities.^20^ Brucea extracts are characterized by complex chemical compositions, and the key components include nigakilactones, alkaloids, triterpenes, steroids, phenylpropanoids, and flavonoids.^21^ A compound in particular, ophiopogonin bitter alcohol, has been extensively studied and has been shown to be potential inhibitors of tumor growth,^22^ but little information has been reported regarding the impact of bruceine D on NSCLC.

Accordingly, in the present study, we investigated the ability of bruceine D to inhibit the growth (Figure 1) and migration (Figure 3) of NSCLC cells. Our results demonstrated that treatment with bruceine D results in a significant inhibition of NSCLC cell proliferation, increases in the levels of cleaved PARP-1 and Bax, and decreases in the level of the antiapoptotic protein Bcl-2. These phenomena are probably caused by the inhibition of STAT3. Therefore, we believe that bruceine D exhibits anti-NSCLC activity (Figure 2).

STAT3 plays important roles in tumorigenesis,^12^ and the inhibition of STAT3 activity has been shown to inhibit the formation of a variety of tumor tissues. In patients with NSCLC, chemotherapy can reduce cancer recurrence and prolong survival in part through a significant reduction in serum interleukin (IL)-6 levels, which leads to decreased activation of STAT3.^23^ High levels of STAT3 phosphorylation have been found to be associated with a variety of cancers.^24^^,^^25^ Furthermore, inhibition of STAT3 has been shown to reduce the growth and metastasis of various tumor types. These observations suggest that targeting STAT3 could be an effective therapeutic strategy for NSCLC. Overall, it is clear that STAT3 can affect the apoptosis, metastasis, immune evasion, and drug resistance of NSCLC, and its tumor-promoting role has been confirmed.^26^ Through additional experiments, we also determined that bruceine D can affect the activity of STAT3. Using molecular dynamics simulation, we found that bruceine D can form hydrogen bonds with four residues in the STAT3 protein, which suggests that bruceine D can bind to STAT3 directly; SPR tests confirmed the ability of bruceine D to bind to STAT3 (Figure 4). In addition, the results of immunoblot analyses showed that bruceine D can inhibit the phosphorylation of STAT3 in a concentration-dependent manner and suppress the expression of downstream proteins (Figure 5).

Despite these strong correlations between STAT3 activity and the development of cancer, no STAT3 inhibitors have been approved for the treatment of cancer. Moreover, because of the clear regulatory relationship between STAT3, MYC, and HIF-1α, inhibiting the STAT3-mediated glycolysis pathway in tumor cells is also one of the new therapeutic strategies to play a role in anti-cancer.^18^^,^^27^ In *in vitro* glycolysis assays, napabucasin has an inhibitory effect comparable to that of bruceine D. Considering that both agents target STAT3 signaling—a critical regulator of glycolytic gene transcription—the comparable magnitude of suppression observed in this metabolic context is mechanistically justified. These observations highlight the link between STAT3 and glycolysis, and our experimental results also confirm the ability of bruceine D to suppress NSCLC glycolysis through STAT3 inhibition (Figure 6).

Previous studies have reported that bruceine D suppresses lung cancer progression through the modulation of the ROS/MAPK^11^ or JNK^28^ pathways, suggesting that its biological activity involves the regulation of oxidative stress and the MAPK signaling network. Emerging evidence indicates a complex, bidirectional regulatory relationship between STAT3 and ROS metabolism; STAT3 participates in maintaining cellular redox homeostasis, and alterations in its activity can influence ROS accumulation and cellular sensitivity to oxidative stress.^29^ Similarly, intricate signaling crosstalk occurs between JNK and STAT3. Notably, studies have shown that the JNK–STAT3–Akt axis cooperatively mediates oncogenic signaling in human bronchial epithelial cells,^30^ with these pathways forming an interconnected regulatory and feedback network. Collectively, these findings provide further theoretical support for the mechanism by which bruceine D inhibits STAT3 and suggest that its antitumor effects may involve multiple interconnected pathways.

Because bruceine D was shown to inhibit STAT3 activity *in vitro*, we further investigated the anti-NSCLC activity of the compound *in vivo*; tests in mouse xenograft models of NSCLC confirmed that the anticancer activity of bruceine D. In these mice, the level of phosphorylation of STAT3 decreased after bruceine D treatment, which supported the model in which the *in vivo* activity of bruceine D was achieved by inhibiting STAT3 activity. In addition, bruceine D also affected levels of the marker of apoptosis, cleaved-PARP1 (Figure 7). Specifically, the results showed that as compared with the control group and with mice treated with the same dose of napabucasin, mice treated with bruceine D experienced a more significant suppressive effect. These findings suggest that, beyond regulating STAT3-mediated glycolysis, Bruceine D may exert additional antitumor effects, potentially involving the modulation of multiple signaling pathways and tumor angiogenesis. Collectively, these data indicate that although both compounds effectively suppress STAT3-driven metabolic reprogramming *in vitro*, Bruceine D may possess broader antitumor efficacy under physiological conditions. Thus, these results have laid the foundation for the clinical application of bruceine D.

In terms of therapeutic efficacy, the IC₅₀ values, apoptosis rates, and related outcomes observed in the present study are generally consistent with those reported previously, although minor discrepancies may be noted. Such differences may be attributable to variations in experimental conditions, sources of reagents, the specific cell lines used for establishing animal models, and other methodological factors.

**Figure 8.**
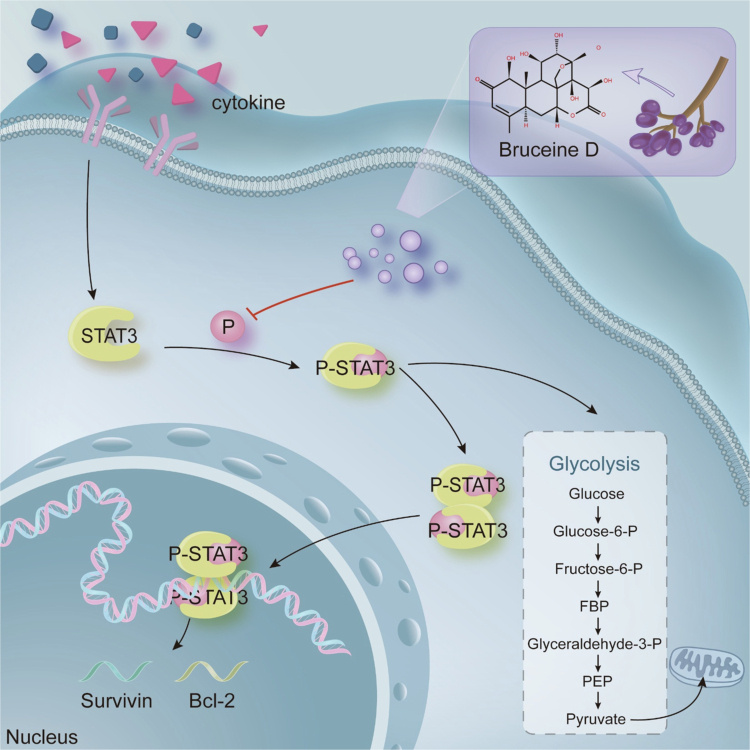
Bruceine D against NSCLC cells achieved by affecting the activity of STAT3.

As an ingredient of traditional Chinese herbal medicine, the low toxicity and high efficiency of bruceine D have often been discussed, and the antitumor ability of bruceine D has been confirmed in previous studies.^28^^,^^31^^,^^32^ Therefore, we propose that the anti-NSCLC activity of bruceine D is related to its effects on the STAT3 pathway (Figure 8).

In conclusion, the present study demonstrates that bruceine D exerts significant inhibitory effects on the growth and metastasis of non-small-cell lung cancer both *in vitro* and *in vivo*, primarily through direct interaction with STAT3 and consequent suppression of its phosphorylation and downstream signaling pathways. These findings reveal a previously unrecognized molecular mechanism underlying the anticancer activity of bruceine D and provide strong experimental evidence supporting its potential as a therapeutic candidate for NSCLC.

Nevertheless, several limitations of this study should be noted. Only three NSCLC cell lines were examined, which may not fully represent the molecular heterogeneity of this disease. Moreover, although the binding between bruceine D and STAT3 was confirmed, the precise binding sites and the detailed mechanisms by which bruceine D regulates STAT3 phosphorylation warrant further investigation. In addition, the dose-limiting toxicity and therapeutic index of bruceine D have yet to be systematically evaluated and should be addressed in future studies.

## Supplementary Material

Supplementary Material Supplementary_Figure_captions.docx

Supplementary Material Supplementary Figure2.tif

Supplementary Material Supplementary Figure1.tif

Supplementary Material Supplementary Figure4.tif

Supplementary Material Supplementary Figure5.tif

Supplementary Material Supplementary_Figure3.tif

Supplementary Material Supplementary Figure6.tif

## Data Availability

All data presented in this article are available by contacting the authors.

## Abbreviations

AP: ammonium persulfate
Bax: Bcl-2-associated X protein
Bcl-2: B-cell lymphoma 2
BD: bruceine D ^1^
DAPI: 4, 6-diamidino-2-phenylindole dihydro-chloride
DMEM: Dulbecco minimum essential medium
DMSO: dimethyl sulfoxide
EdU: 5-ethynyl-2′-deoxyuridine
FBS: fetal bovine serum
GAPDH: glyceraldehyde 3-phosphate dehydrogenase
HE: hematoxylin–eosin staining
IC_50_: the half-maximal inhibitory concentrations
IL-6: Interleukin 6
MTT: methylthiazolyldiphenyl-tetrazolium bromide
NSCLC: non-small-cell lung cancer
OD: optical dens
PBS: phosphate-buffered saline
PI: propidium iodide
PVDF: polyvinylidene fluoride
SD: standard deviation
SDS-PAGE: sodium dodecyl sulfate-polyacrylamidegel
STAT: signal transducer and activator of transcription
Rot/AA: rotenone and antimycin A mixture
2-DG: 2-Deoxy-D-arabino-hexose
