# Supplementary material for: Inhibition of STAT3-mediated glycolysis by bruceine D suppresses non-small-cell lung cancer progression in vitro and in vivo
Source: Cancer Biol Ther. 2026 May 8;27(1):2665867. doi: 10.1080/15384047.2026.2665867 (PMC13166240; doi:10.1080/15384047.2026.2665867)
Supplement: Supplementary Material — Supplementary_Figure_captions.docx [file KCBT_A_2665867_SM3735.docx]

**Supplementary Figure 1. Bruceine D induced migration in NSCLC cells**

(A) Survival rate of BEAS-2B cells under different concentrations of bruceine D treatment (B)After treated with bruceine D for 24 h, EdU reagent was added to detect the proliferation of PC-9, SKMES-1(100× magnification). (**P*<0.05, ***P*<0.01, ****P*<0.001, *****P*<0.0001.)

**Supplementary Figure 2. Bruceine D induced migration in NSCLC cells**

Statistical graph of Western blot analysis of various apoptosis-related proteins in different NSCLC cell lines. (**P*<0.05, ***P*<0.01, ****P*<0.001, *****P*<0.0001.)

**Supplementary Figure 3 Bruceine D induced migration in NSCLC cells**

Statistical graph of Western blot analysis of various migration-related proteins in different NSCLC cell lines. (**P*<0.05, ***P*<0.01, ****P*<0.001, *****P*<0.0001.)

**Supplementary Figure 4 Bruceine D blocks the activation of STAT3**

(A) Statistical graph of Western blot analysis of P-STAT3, Survivin in different NSCLC cell lines. (B) Statistical graph of Western blot analysis of STAT3 in nucleus and cytoplasm. (**P*<0.05, ***P*<0.01, ****P*<0.001, *****P*<0.0001.)

**Supplementary Figure 5. Bruceine D blocks STAT3 entry into the nucleus**

Microscopic images indicating the localization of P-STAT3 (green) and DAPI in SKMES-1 cells (600× magnification).

**Supplementary Figure 6. Bruceine D inhibited the glycolysis of NSCLC.**

(A) NSCLC cells were treated with bruceine D for 48 h, detection of glucose consumption in control or bruceine D group. (B) NSCLC cells treated with bruceine D for 48 h, detection of lactic production in control or bruceine D group. (C) PC-9 treated with bruceine D or napabucasin for 48 h, detection of glucose consumption and lactic production in control or treatment group. (D) When STAT3 is overexpressed, PC-9 treated with bruceine D (2.5μM) for 48 h, detection of glucose consumption and lactic production in control or treatment group.
