# Supplementary figures and images for: Inhibition of STAT3-mediated glycolysis by bruceine D suppresses non-small-cell lung cancer progression in vitro and in vivo
Source: Cancer Biol Ther. 2026 May 8;27(1):2665867. doi: 10.1080/15384047.2026.2665867 (PMC13166240; doi:10.1080/15384047.2026.2665867)

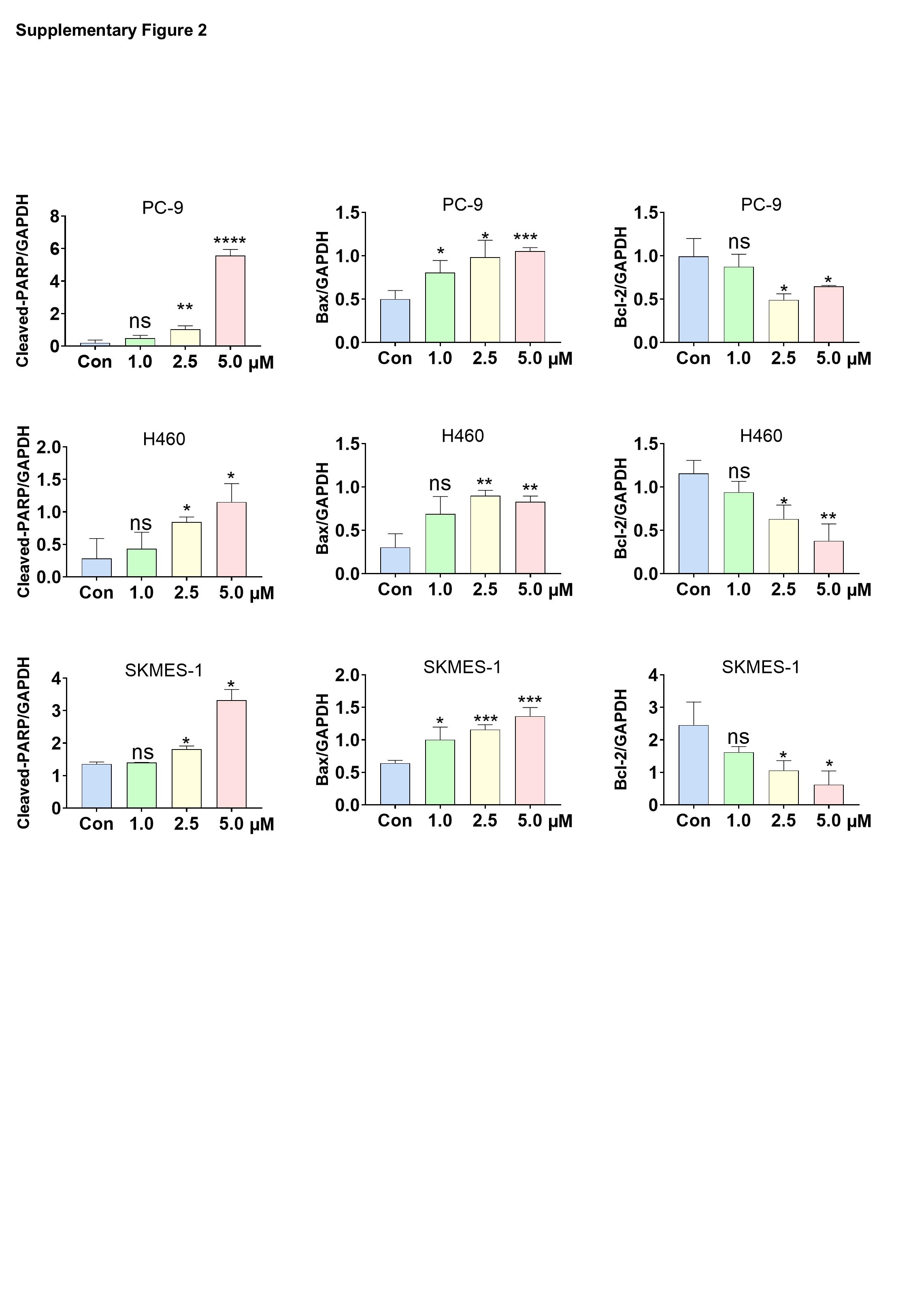

Supplement: Supplementary Material — Supplementary Figure2.tif [file KCBT_A_2665867_SM2952.tif]

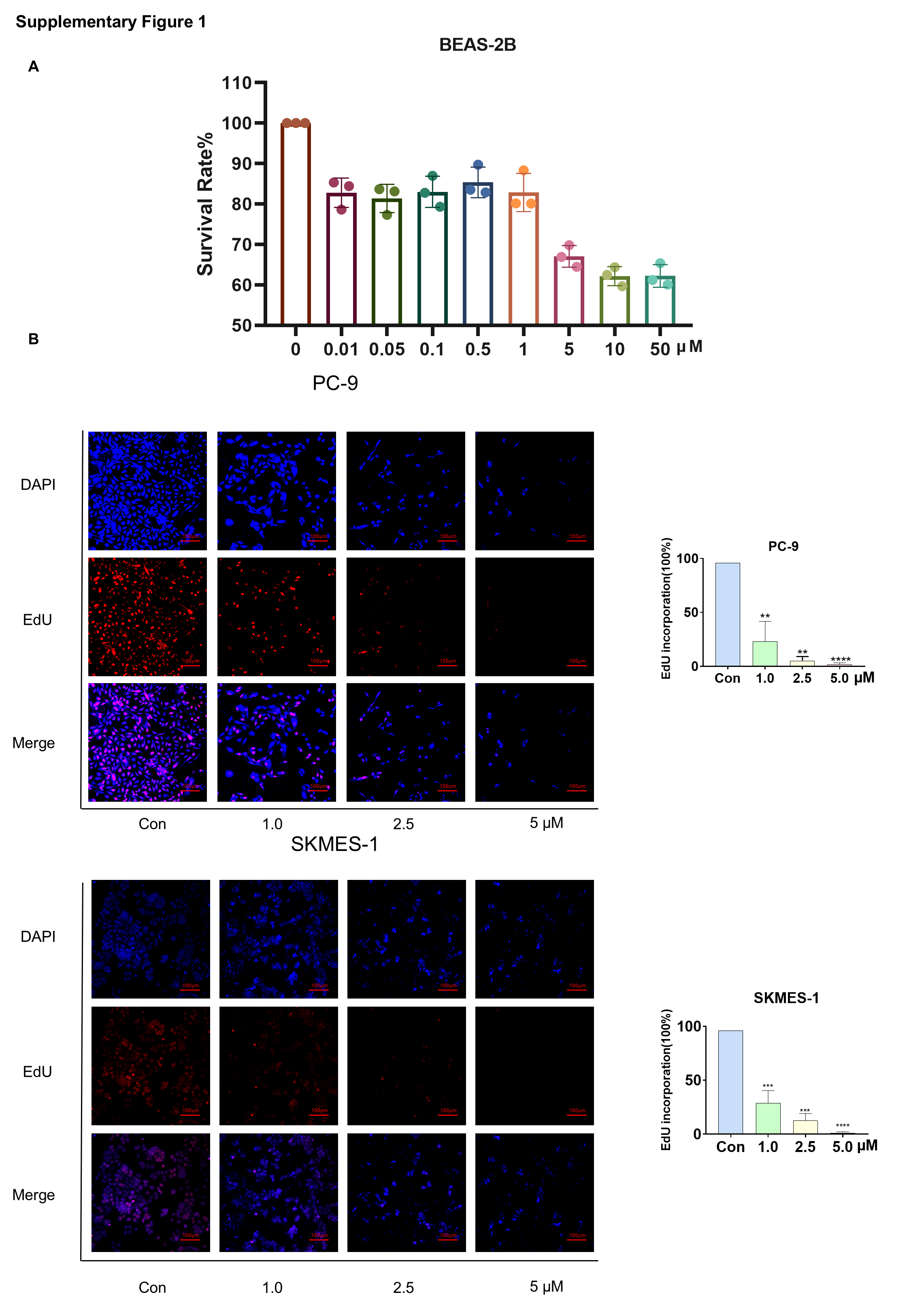

Supplement: Supplementary Material — Supplementary Figure1.tif [file KCBT_A_2665867_SM2951.tif]

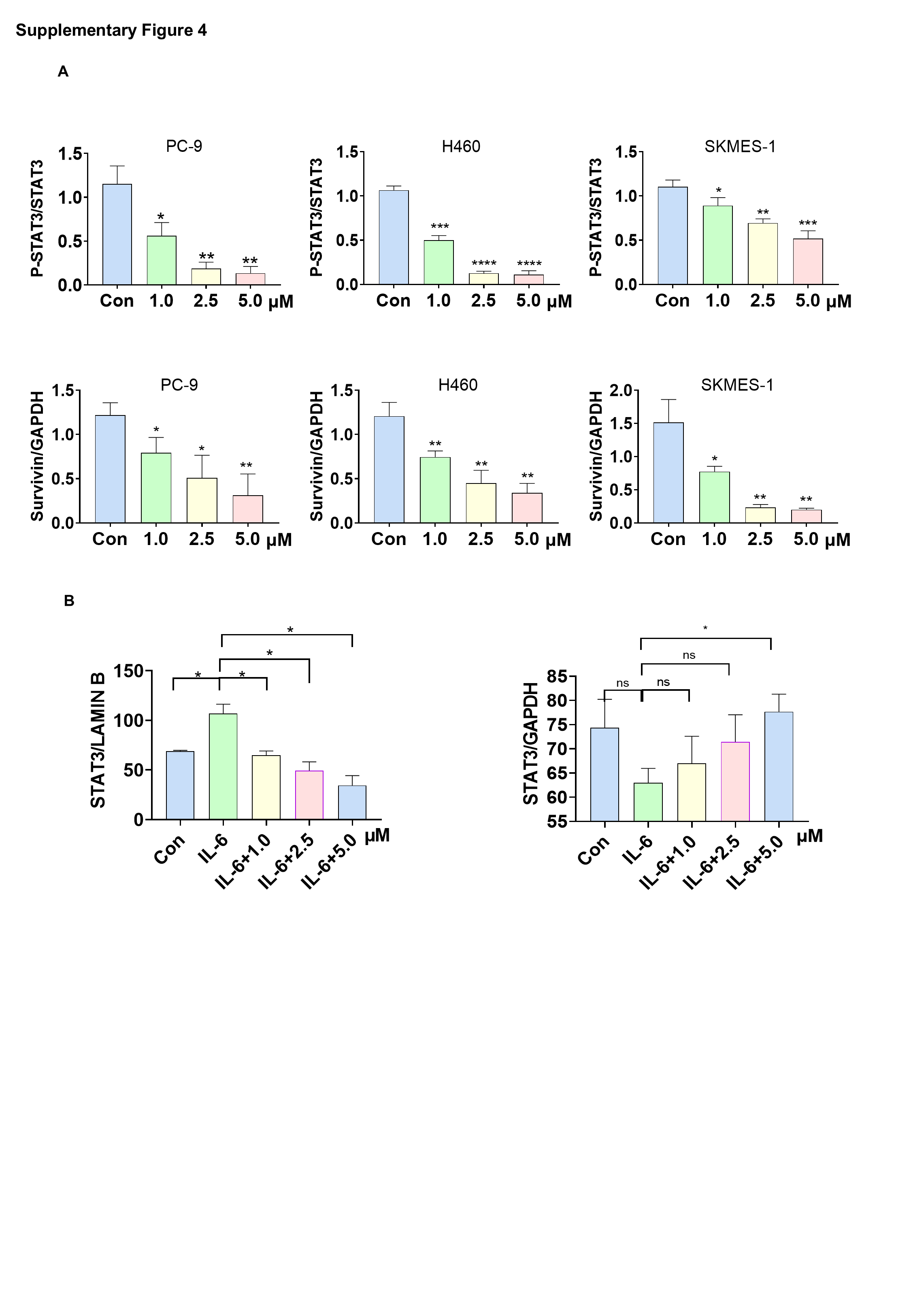

Supplement: Supplementary Material — Supplementary Figure4.tif [file KCBT_A_2665867_SM2949.tif]

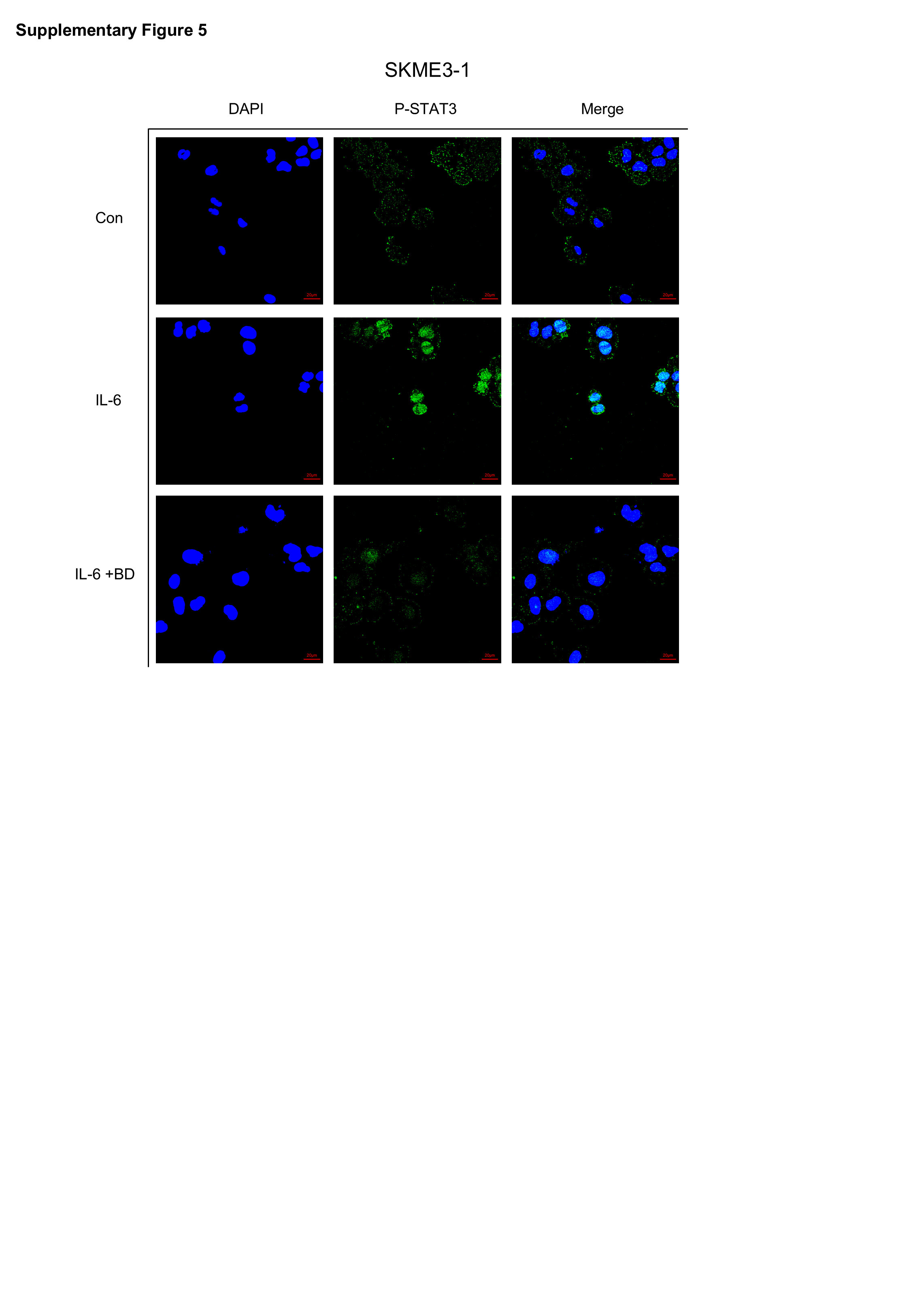

Supplement: Supplementary Material — Supplementary Figure5.tif [file KCBT_A_2665867_SM2948.tif]

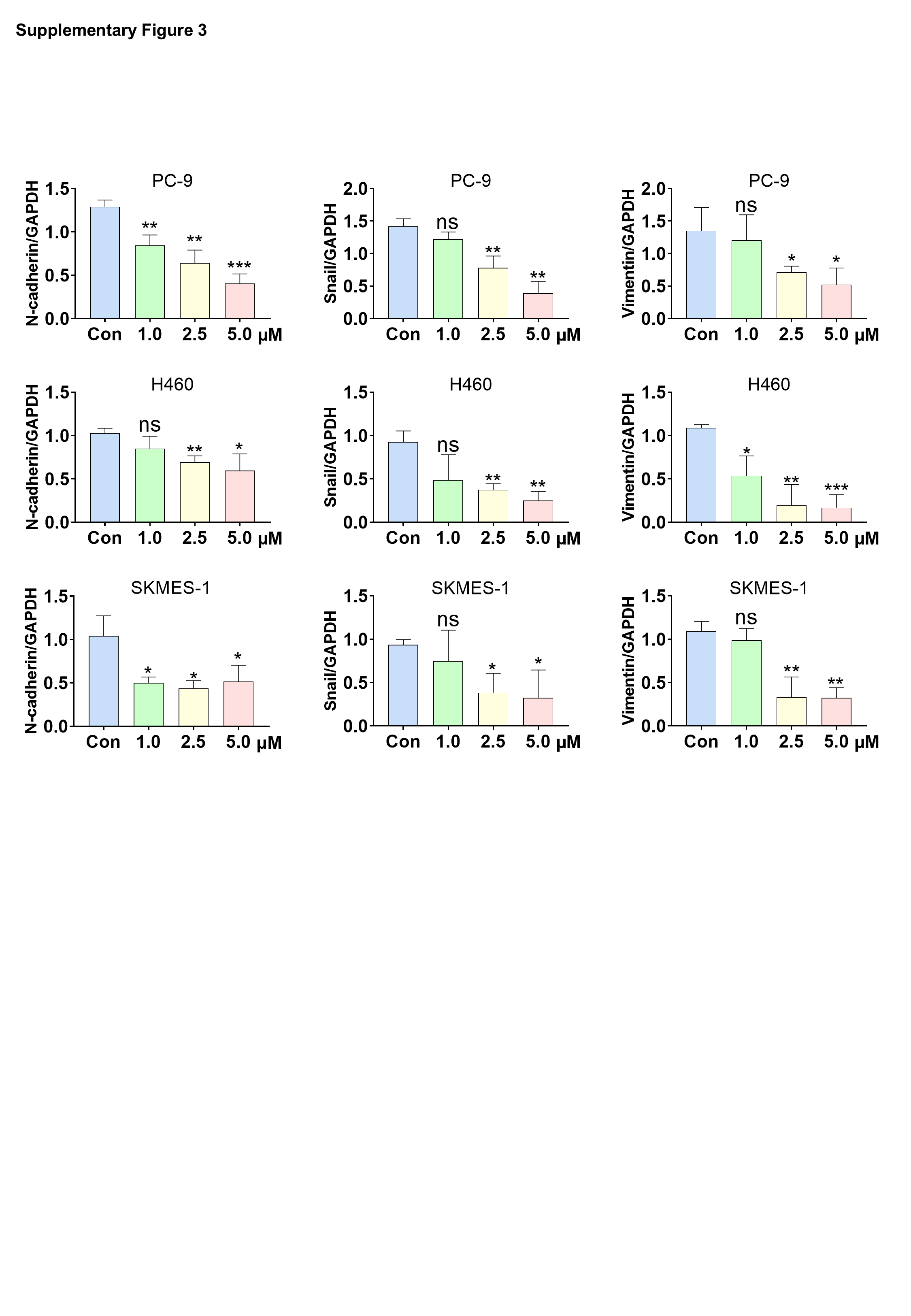

Supplement: Supplementary Material — Supplementary_Figure3.tif [file KCBT_A_2665867_SM2947.tif]

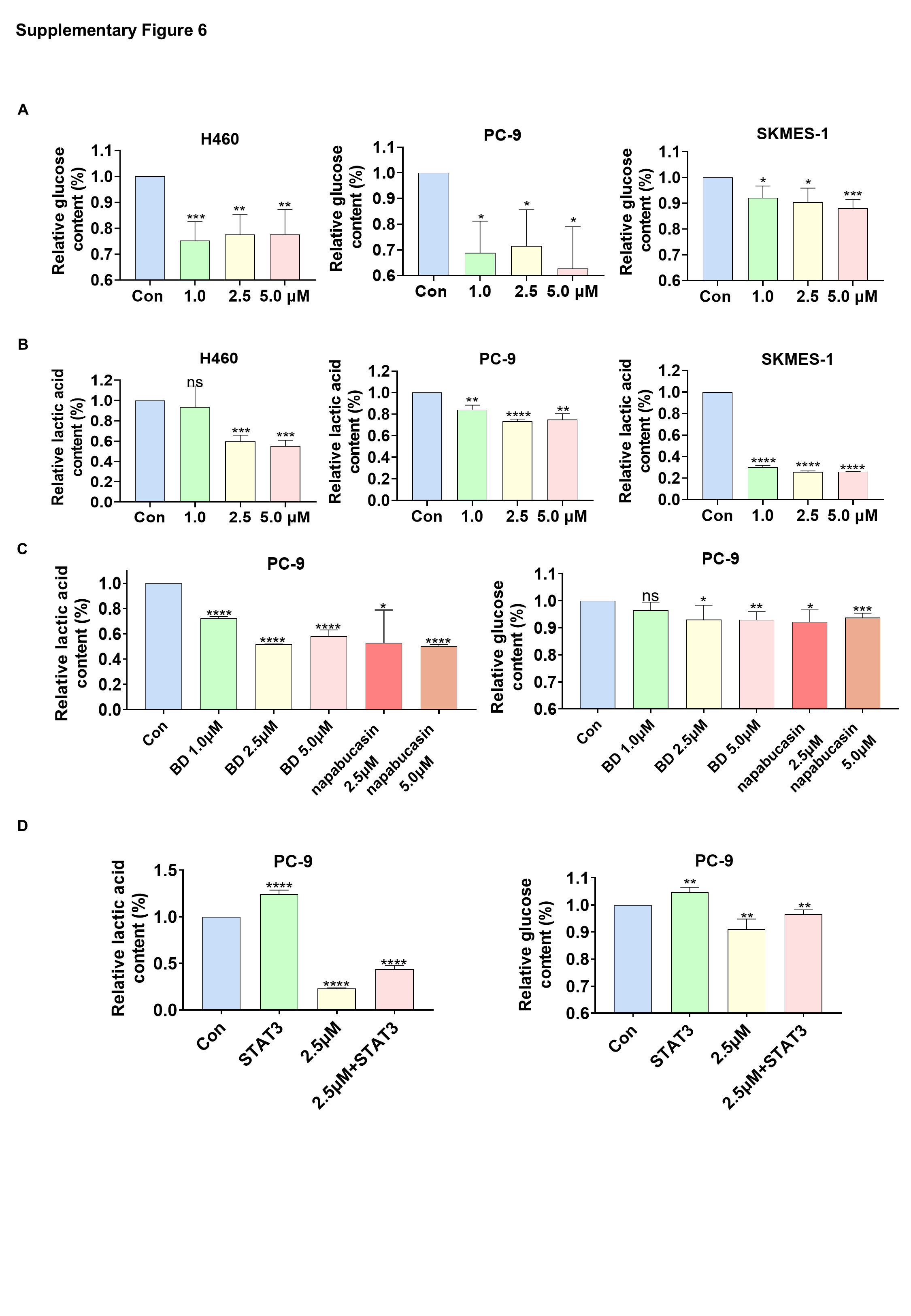

Supplement: Supplementary Material — Supplementary Figure6.tif [file KCBT_A_2665867_SM2946.tif]
